# Reply to “Misclassification of PfEH1 and PfEH2 as Epoxide Hydrolases”

**DOI:** 10.1128/mBio.00235-17

**Published:** 2017-03-07

**Authors:** Daniel E. Goldberg, Natalie J. Spillman

**Affiliations:** a Departments of Medicine and Molecular Microbiology, Washington University School of Medicine, St. Louis, Missouri, USA; b Research School of Biology, The Australian National University, Canberra, Australian Capital Territory, Australia; NIAID/NIH

## REPLY

In our recent study, we characterized two exported proteins, PfEH1 and PfEH2, of *Plasmodium falciparum* (1). Overexpression of these proteins resulted in a decrease in epoxide-containing lipids in infected erythrocytes, which we proposed was due to the epoxide hydrolase activities of these α/β hydrolases. We appreciate the thoughtful comments of Arand and Marowsky (2), who propose an alternative explanation of our data: that PfEH1 and PfEH2 are esterases.

The actions of the recombinant enzymes on the epoxy fluor 7 (EF7) substrate may certainly be due to esterase activity. However, the activity against the purified epoxide-containing lipids (EETs) is harder to discount. Hydrolysis was low in this nonoptimized assay but was reproducible and statistically significant. We would not expect even this low level of hydrolysis if an ether were being formed. We are aware of, and refer in the Discussion section of our article (1), to the stable reaction intermediate formed from serine nucleophile action on an epoxide; an alternative in the case of the *P. falciparum* enzymes is that another residue is involved, perhaps through activation of water for nucleophilic attack. Determining the crystal structure of PfEH1/2 would assist in examining this hypothesis.

It is certainly possible that the epoxidase activity that we propose plays biological second fiddle to the potential esterase activity suggested by Arand and Marowsky. Further experiments to study the full substrate range of the recombinant enzymes and how this is physiologically relevant in infected erythrocytes are needed.
